# Can fluoxetine mitigate mental health decline in musculoskeletal trauma patients: a pilot single-center randomized clinical trial

**DOI:** 10.1186/s40814-022-01119-8

**Published:** 2022-08-17

**Authors:** Elizabeth Lossada-Soto, Marissa Pazik, Mary Beth Horodyski, Terrie Vasilopoulos, Ludmila Barbosa de Faria, Carol Mathews, Jennifer Hagen

**Affiliations:** 1 Department of Orthopaedic Surgery & Sports Medicine, University of Florida, PO Box 112727, Gainesville, FL 32611-2727 USA; 2 Department of Anesthesiology, University of Florida, 1600 SW Archer Road, Gainesville, FL 32610 USA; 3 Department of Psychiatry, University of Florida, 1149 Newell Drive, Suite L4-100, Gainesville, FL 32610 USA

**Keywords:** Orthopedic trauma, Mental health, Anxiety, Depression, Post-traumatic stress disorder (PTSD), Fluoxetine, Prozac

## Abstract

**Background:**

Musculoskeletal trauma is one of the leading causes of disability in the USA and its negative quality of life impact extends beyond that of physical recovery. More than 50% of victims of musculoskeletal trauma suffer lasting mental health issues and post-traumatic stress disorder (PTSD) symptomology following their injury. These symptoms can develop across all spectrums of patients and are independent predictors of poor outcome. Access to mental health care is limited, expensive, and time intensive, and a large majority of the trauma population do not get to utilize this valuable resource. This leaves the burden of management on the orthopedic team, as they are often the only point of contact for the patient within the medical system.

**Methods:**

This is a single-center, repeated measures, randomized controlled pilot study including up to 100 orthopedic trauma patients aged between 18 and 85 years of age. Subjects are approached during their index hospitalization and are randomized to one of two pharmaceutical interventions, fluoxetine (also known as Prozac) or calcium, for 9 months. Fluoxetine is a selective serotonin reuptake inhibitor (SSRI) that is supported for the treatment of PTSD by the American Psychiatric Association. It is low-cost and has minimal side effects and withdrawal symptoms if stopped suddenly. Calcium is a supplement with minimal side effects that is used in our study for its bone-healing potential. Feasibility will be indexed by recruitment feasibility, randomization feasibility, medical adherence, anti-depressant side effects, and fracture union rate. Subjects will complete physical and mental health surveys at baseline, 2 weeks, 6 weeks, 3 months, 6 months, and 1 year.

**Discussion:**

The goals of this exploratory clinical trial are to: develop a safe, feasible, and time-limited protocol effect of immediate (post-injury) treatment with fluoxetine for use by orthopedic providers and other non-mental health care providers treating victims of musculoskeletal trauma (Aim 1), and test the for preliminary effects of the protocol on development of PTSD symptomology and physical recovery in these patients (Aim 2). This study is novel in that it strives to prevent the development of symptomology from the time of injury and empowers surgeons to manage their patients in a more holistic manner.

**Trial registration:**

ClinicalTrials.gov, NCT04850222. Registered on April 20, 2021.

## Background

The negative life impact of musculoskeletal trauma victims goes beyond physical recovery. In addition to being one of the leading causes of disability in the USA, musculoskeletal trauma also leaves more than 50% of victims with lasting mental health issues post-injury [1]. Mental health problems are the largest cause of disabilities worldwide [2]. Prolonged depressive and anxious symptomology following a traumatic injury is an independent risk factor for prolonged physical disability. This symptomology can develop across a wide spectrum of patients with a variety of different injury severities. Previous studies have attempted to use non-medication interventions to prevent these negative symptoms [3]. In 2015, the UF orthopedic trauma division developed a ten-step program to help victims of musculoskeletal trauma develop coping mechanisms and self-activation to improve both physical function and mental health scores in the year following trauma. While the program was well received by patients and healthcare providers alike, we were unable to demonstrate a measurable benefit to either physical or mental wellbeing scores [3]. This unfortunately has been mirrored in other studies: an investigation into the use of formal cognitive behavioral therapy (CBT) in patients with hip fracture did not demonstrate any improvement in well-being measures [4]. CBT did show some efficacy at lessening symptoms of PTSD in meta-analysis performed on studies of patients with multisystem trauma, but only when guided in person by a provider, not when given via a self-help booklet or an internet CBT intervention [4].

Access to trained mental health care providers is a scarce, valued resource, which has become increasingly stressed worldwide. Models have been established that empower primary care providers and oncologists to initiate medical management for patients exhibiting signs of depression, anxiety, and post-traumatic stress disorder [5, 6]. Studies examining the utilization of mental health resources following mass-traumatic events in the civilian population demonstrate that access to and utilization of mental health resources are low, drop-out rates are high and more than 50% of those who are able to obtain and keep care, utilize psychopharmacological treatment [7]. This leads to the purpose of this pilot study. Our goal is to develop a safe, feasible, and time-limited treatment strategy that could be safely implemented by non-mental health care providers for victims of musculoskeletal trauma. We hypothesize that a stronger, pharmaceutical intervention started after surgery and prior to severe depressive and anxious symptoms can diminish negative mental health symptoms in orthopedic trauma patients.

## Methods/design

### Aims

- Primary aim (Aim 1): develop a safe fluoxetine treatment protocol with a goal to be titrated to a standardized dose by 6 weeks and taper completely off treatment by 6–12 months.
  - ° *Hypothesis:* we will be able to develop a standardized, time-limited protocol that could feasibly be utilized by non-mental health care providers and tolerated by patients.
  - ° *Methodology:* we will measure medication adherence, medication toleration and side effects, fracture union rate, and other feasibility metrics in the 12-month recovery period.
- Secondary aim (Aim 2): evaluate the efficacy of immediate fluoxetine therapy versus calcium in improving PTSD symptomology and physical and mental recovery in the post-injury period for patients with musculoskeletal trauma.
  - ° *Hypothesis:* patients randomized to fluoxetine will have less severe depression, anxiety, and post-traumatic stress symptoms, and improved self-reported functional scores, than those receiving calcium.
  - ° *Methodology:* patients will be randomized to fluoxetine or calcium during hospitalization following their trauma. Serial patient reported outcomes measures and mental health surveys (BDI-II-Beck Depression Inventory-II; BAI-Beck Anxiety Inventory; PSS-SR5-PTSD Scale-Self report for DSM-5) will be obtained prior to the initiation of treatment and during the first 12 months of their recovery to assess severity and duration of PTSD symptomology

### Design

This is a single-center, repeated measures, non-blinded randomized controlled pilot study. A maximum of 200 participants will be involved in the study, ranging in age from 18 to 85 years old. This is an investigator-initiated trial that was registered with ClinicalTrials.gov (NCT04850222) on April 20, 2021 before patient enrollment began in September 2021. All study procedures have been approved by the University of Florida’s Institutional Review Board for the Protection of Human Subjects. All research team members have been trained in Good Clinical Practice for Clinical Trials. The financial sponsor of this study is the Orthopaedic Trauma Research Fund at the University of Florida.

#### Design rationale

- *Fluoxetine:* fluoxetine, commonly known as Prozac, is an antidepressant that works as a selective serotonin reuptake inhibitor (SSRI) [8]. It has typically been prescribed for depression and may also help with anxiety symptoms, with minor to no side effects. Our team chose fluoxetine versus other antidepressants due to its lack of severe side effects, positive outcomes particularly in PTSD patients, and its familiarity in the general population [9].
- *Calcium:* calcium is a supplement that may provide some bone healing potential, but overall should have minor side effects on the patients [10]. Our team chose calcium because we wanted both study arms to potentially receive a health benefit, whether mental (fluoxetine) or physical (calcium). Utilizing a placebo would not provide an additional health benefit. We selected elemental calcium in particular because most calcium formulations include vitamin D to help with calcium absorption, but studies have shown vitamin D may have mental health benefits [11]. We want to avoid directly impacting the mental health outcomes of the “non-mental health drug” study arm.
- *Non-blinded:* our team has decided to completely unblind for two reasons. First, for patient safety, we prefer our research and clinical team to be aware of a patient’s randomized medication to prevent and better prepare for any adverse side effects. Secondly, we believe that patients that may be biased or hesitant to take a mental health medication will feel more comfortable knowing what medication they are randomized to.

### Locations

Our patients will only be recruited at UF Health at Shands Hospital located in Gainesville, FL, USA. This is a level 1 trauma center serving 18 urban and rural counties comprising 2 million people. The hospital admits approximately 4500 patients with traumatic injuries annually. Informed consent and baseline surveys were collected at the level 1 trauma center. After discharge, patients are followed up with at the University of Florida Orthopaedic and Sports Medicine Institute. This facility houses the outpatient orthopedic clinics and is where all patients receive their follow-up care and study subjects will receive their surveys unless phone or email follow-up is required.

### Research team

Our research team includes our Principal Investigator Dr. Jennifer Hagen, Division Chief and Associate Professor of Orthopaedic Trauma. Co-Investigators include Dr. Carol Mathews, Professor and the Vice Chair for Strategic Development in the Department of Psychiatry, Dr. Ludmila Barbosa de Faria, Associate Professor in the Department of Psychiatry, Dr. MaryBeth Horodyski, Professor and Director of Research in the Department of Orthopaedic Surgery and Sports Medicine, and Dr. Terrie Vasilopoulos, Assistant Professor of Anesthesiology and Orthopaedic Surgery and Sports Medicine. Additionally, all five orthopedic trauma surgeons and five Physician Assistants gained IRB approval and assisted in study procedures. Drs. Hagen and Barbosa de Faria review and report serious adverse events (SAEs), escalation of mood disturbance, and drug side effects in real time. Dr. Carol Mathews reviews study conduct and data and provides recommendations as needed. Research study team members (including one Clinical Research Coordinator and one Research assistant) are involved in subject recruitment and consent administration, in-person and phone call study follow-ups, and day-to-day administration work.

### Patients

#### Inclusion criteria

- Persons aged 18–85 years old
- Admitted for high energy musculoskeletal trauma (more than ground level fall) resulting in one or more operative extremity or pelvic fractures
- Have granted informed consent and enrolled for the study in the hospital prior to discharge.

#### Exclusion criteria

- Presence of a traumatic brain injury
- Past medical history or self-reported bipolar disorder or other mental health conditions on active pharmacologic treatment (including but not limited to suicidal ideation or depression)
- Current use of mood stabilizers and atypical antipsychotics
- Pregnancy
- Incarceration
- Expected injury survival of less than 90 days
- Unlikely to maintain follow-up
- Any contra-indications to fluoxetine or calcium
- Unable to provide informed consent due to language barriers or other barriers (e.g., intubation)
- Fracture managed outside of the participating orthopedic service (e.g., a hand fracture managed by plastics)
- Transferred from an outside hospital
- Have a medical or physical condition in opinion of the investigator that would preclude safe participation in the study.

### Recruitment and consent

Study recruitment began in September 2021. Planned enrollment for race, ethnicity, and sex can be found in Table 1. After admission to UF Health Center and after at least one orthopedic surgical intervention, patients are approached by a study team member who has pre-screened the patient for potential recruitment. Before initiating the informed consent process, the study team member assures that the patient is stable enough to proceed with consenting. Standard IRB enrollment guidelines are followed by team members to properly consent and enroll participants.

**Table 1:** Study planned enrollment report (based on previous prospective study in this patient population) [1]

Racial categories	Ethnic categories				
	Not Hispanic or Latino		Hispanic or Latino		Total
	Female	Male	Female	Male	
**American Indian/Alaska Native**	1	1			**2**
**Asian**	1	1			**2**
**Native Hawaiian or other Pacific Islander**	1	1			**2**
**Black or African American**	4	10			**14**
**White**	30	43	4	3	**80**
**More than one race**					**0**
**Total**	**0**	**0**	**0**	**0**	**100**

Personal subject data such as name, medical record number, age, home address, and phone number(s) will be de-identified as each subject is assigned a research identification number after consenting. This is to assure that at the completion of the study, no one will be able to associate the patient’s personal information with their study data. The master link between their research identification number and personal identifiers will be kept in a secure location and accessible to study personnel only. Additionally, this study team has obtained a Certificate of Confidentiality from the National Institutes of Health to further protect the subject’s privacy. Study data will be reviewed on a quarterly basis by Dr. Mathews and no external site monitor will be used for this study.

### Survey administration

The initiation of the study occurs at baseline, which is after the patient has been consented, but prior to hospital discharge and initiation of the randomized study medication. At baseline, patients begin with surveys regarding their depression, anxiety, a self-report of mental health concerns, and pain, enjoyment, and activity scales (Table 2). As it is the participant’s first time hearing the questions, these surveys may take up to 30 min. After hospital discharge, patients return to the orthopedic clinic for post-surgical follow-ups. To minimize patient burden, study data is collected during their standard of care clinic follow-up visits. This means subjects will not need to visit the clinic for study purposes only and will come into the clinic as often as a non-study participant patient. Study personnel will collect patient data and administer surveys at follow-up visits at 2 weeks, 6 weeks, 3 months, 6 months, and 12 months (1 year) post-surgery. To maintain quality follow-up rates, phone surveys will be planned as the type of follow-up for the 1-year visit, unless the patient reports surgery-related issues and is present in the clinic for a scheduled visit. The survey schedule for each visit can be found in Table 2. Total time to complete all the surveys at each timepoint is 15–20 min. Follow-up visits can be within a given range to meet the timeframe criteria, and this range for each follow-up is noted in Table 2.

**Table 2:** Outcome assessment schedule

	Baseline	2 weeks	6 weeks	3 months	6 months	1 year
**Enrollment**						
Eligibility	**x**					
Consent	**x**					
Demographics	**x**					
**AIM 1**						
***Feasibility in Recruitment, Randomization, and Retention***						
Counts for screened, eligible, and refusal (along with reasons)	**x**					
Counts for consents and randomization refusals	**x**					
Counts for number of completed follow-up visits		**x**	**x**	**x**	**x**	**x**
***Medication adherence, safety, and acceptability***						
MMAS-8		**x**	**x**	**x**	**x**	**x**
Desire to continue medication					**x**	**x**
ASEC			**x**	**x**	**x**	**x**
**AIM 2**						
***Mental health***						
BDI-II	**x**	**x**	**x**	**x**	**x**	**x**
BAI	**x**	**x**	**x**	**x**	**x**	**x**
PSS-SR5	**x**	**x**	**x**	**x**	**x**	**x**
***Sleep quality***						
PSQI		**x**	**x**	**x**		
***Pain and pain interference***						
PEG	**x**	**x**	**x**	**x**	**x**	**x**
***Social participation***						
PROMIS			**x**	**x**	**x**	**x**

*BDI-II* Beck Depression Inventory-II, *BAI* Beck Anxiety Inventory, *PSS-SR5* PTSD Scale-Self report for DSM-5, *PSQI* Pittsburgh Sleep Quality Index, *PEG* Pain, Enjoyment, General Activity Scale, *PROMIS* physical function, *MMAS-8* Morisky Medication Adherence Scale, *ASEC* Antidepressant Side-Effect Checklist

In addition to collecting patient reported outcomes we will also record use of narcotics, cannabis supplements, illegal substances, and alcohol consumption. Prescription drug monitoring program (PDMP) will be queried for other sources of narcotic prescriptions. Patient reported pain scores (PEG score) will be recorded at each visit as one of the required surveys for completion. Any complications related to the surgery (e.g., infection, non-union, unplanned return to operating room) will be documented. If patients do not return to clinic for their follow-up appointment, study personnel will call the patients to complete the surveys over the phone or send the participant the survey via email.

#### Drug administration and cessation

Patients will be made aware of what drug they are randomized to after the informed consent process and baseline surveys are completed. They will begin their randomized study medication before hospital discharge. While the patient is still admitted in the hospital, they will be prescribed a 90-day supply of the randomized medication to take post-hospital discharge. Those patients that have been randomized to fluoxetine will be started on 10mg daily and have their dose increased to 20 mg PO/day at their 2-week visit, while the calcium group will continue their 1000 mg original dose. Patients will be provided with subsequent 90-day prescriptions by our outpatient pharmacy at 3 months and prescriptions will be billed to the study. At the 6-month visit, one last 90-day prescription of the participant’s randomized medication will be given. If patients are unable to come into the clinic for this visit, their medication will be mailed to them. If patients are unable to come into the clinic for any of their visits within the required timeframe for survey completion, they will be contacted via phone call or email to complete the required surveys. Study personnel will use the phone numbers and emails provided by patients at baseline, and any phone or email changes will be updated at each clinic visit to assist with subject follow-up success.

**Fig. 1:**
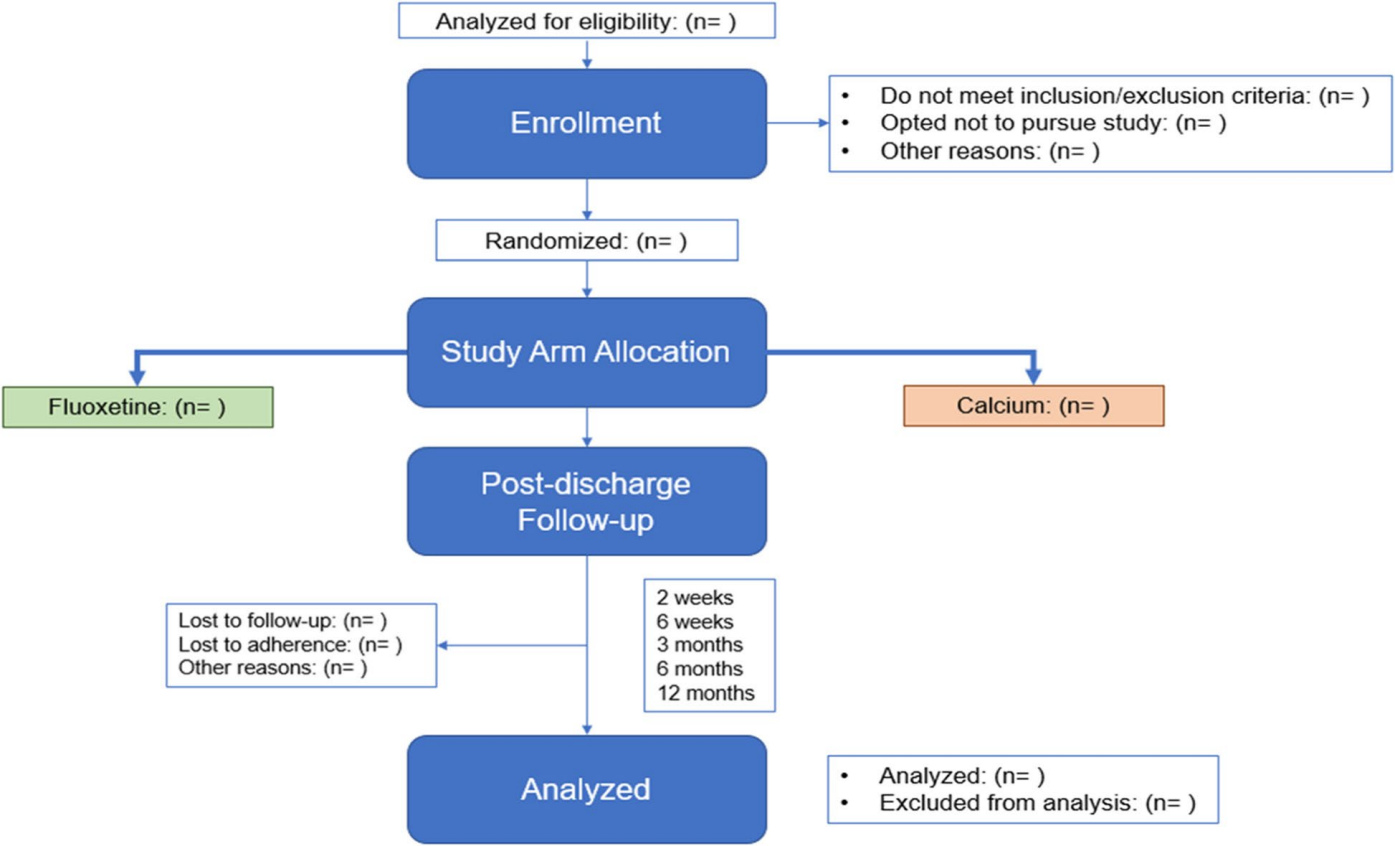
Study flow diagram

**Fig. 2:**
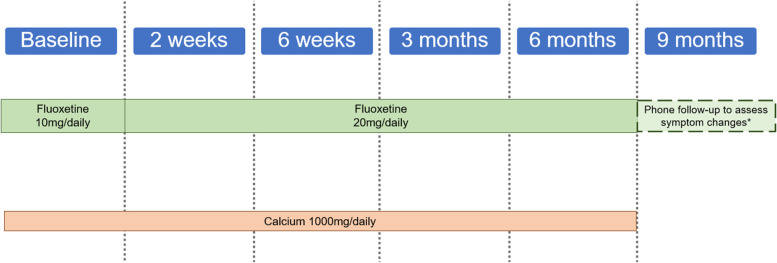
Drug administration timeline (*Referral to primary care physician if needed for further mental health treatment)

Although there is no survey requirement at 9 months post-surgery, patients will receive a follow-up phone call or email around this timeframe to check in on their post-medication cessation symptoms. Patients will be followed for 3 months after the cessation of drug therapy to monitor for worsened symptomology. If they have continued or worsened mental health symptoms, they will be referred for further evaluation. A visual of the study timeline is shown in Fig. 1, and a visual of the drug administration timeline is provided in Fig. 2.

### Study retention

Retaining patients in all 12 months of the study period will be a priority of the study team. To avoid loss to follow-up, patients that are unable or unwilling to return to the clinic for in-person appointments will be contacted via phone call. When feasible and appropriate, patients may also be offered telemedicine appointments. Phone surveys will be planned as the type of follow-up for the 1-year visit, unless the patient reports surgery-related issues and is present in the clinic for a scheduled visit.

### Safety monitoring

A monthly review will be conducted for participant side effects, mood scores, and medication adherence by Dr. Hagen and Dr. Barbosa de Faria. Any serious adverse event (SAE) will be identified and addressed, and properly adjusted through dose titration, medication stoppage, and/or mental health evaluation. If any participant answers a survey question that exceeds a threshold where they may be experiencing harmful thoughts, questionnaires have been set up in REDCap to alert Dr. Barbosa de Faria and Dr. Hagen. The thresholds for alerting the study team vary by survey and are established as follows: BDI II more than or equal to 20 (moderate range); BAI = more than or equal to 26 (severe range); PSS SR: anything above 34 (moderate range). With this notification, Dr. Barbosa De Faria and Dr. Mathews will triage issues, and appropriate care will be given. Participants may be referred to UF Health mental health services or a local community mental health organization.

### Potential discomfort and risks

All participants for the study have been admitted to the hospital through orthopedic surgery to treat their injury. There is no additional anesthetic or complication risk that is subjected to study participants that is not already a typical risk for patients following orthopedic surgery. These risks are discussed with the patient prior to surgery and are not directly related to the study. Many of the surveys presented in the study may be uncomfortable or even distressing for some patients. The questions in the surveys regard their pain, mood, and feelings and include distressing themes such as self-harm and suicide. All answers are confidential and only viewed by the IRB approved study staff. If patients state that they are expressing self-harm or thoughts of suicide, study personnel will address them as specified prior. This study may include risks that are unknown at this time.

- Risks related to study drugs are documented below:
  - ▪ Fluoxetine: some patients taking fluoxetine might experience GI distress (nausea, vomiting, diarrhea), headaches, insomnia, nervousness, anxiety, sweating, abnormal dreams, and dizziness. If patients experience these symptoms, then Dr. Barbosa de Faria or Dr. Mathews will decrease the dose or stop the medication. It is possible patients could have improvement in depressive, anxious, and other negative mood symptoms if they are randomized to fluoxetine. As fluoxetine has a decades long safety record in the general population, we do not anticipate having to stop the trial due to overwhelming participant side effects. If an individual patient is unable to tolerate fluoxetine, they will no longer be given the medication but will be followed for the duration of the study (12 months) and analyzed as intent-to-treat.
  - ▪ Calcium: some patients taking calcium might experience some minor side effects such as belching or gas. Calcium is potentially unsafe when taken by mouth in doses above the daily tolerable upper intake level (UL). The UL is 2000 mg for adults ages 19 and older and 1300 mg for adults aged 18. Our dosage of calcium for subjects in the calcium group is 1000 mg by mouth per day, well within the safety margins. Taking more than this amount of calcium daily can increase the chance of having serious side effects, such as milk-alkali syndrome, a condition that can lead to renal stones, kidney failure, and death. It is possible patients in the calcium group could benefit from calcium’s bone healing potential.

### Randomization

#### Allocation and concealment

- Once consented, patients will be randomized to fluoxetine (10 mg by mouth per day) or calcium (1000 mg by mouth per day). The randomization will be stratified by gender and initial BDI-II score (no/mild symptomology BDI 0–13 or moderate severe symptomology BDI > 14). Both BDI-II groups will be randomized to treatment because although the initial BDI-II was correlated with long-term symptomology, the prior study also had patients with no initial symptomology develop PTSD over time [1]
- Medication randomization was done before beginning the consenting process, and stringent measures have been taken to prevent bias. The established order of medication randomization will be stored in two places: a secured online drive and within sealed envelopes in a secure location. To prevent bias, the envelopes are used to hide the next randomized medication from the view of the orthopedic team member who administered the consent. The envelopes are separated by BDI score and gender (BDI < 13 or > 14, male or female). After consent has been given and the baseline BDI survey administered, the orthopedic team member will select the envelope that corresponds to the patient to reveal the randomization group. The randomized medication will be prescribed by the orthopedic team on the day of randomization to enhance monitoring of the patient for side effects during the remainder of their hospitalization. The patient will be prescribed the randomized medication on the day of discharge with a 90-day supply provided by the inpatient research pharmacy.

### Outcome measures

#### Aim 1: feasibility and safety

Feasibility metrics will be collected for recruitment, randomization, and retention. We will obtain counts for number of patients screened, eligible, and refused, as well as reasons for refusal. We will also assess counts of numbers of patients who consent and who refuse their randomization. Finally, because it is a longitudinal study, we will collect retention and drop-out rates at each follow-up. We will be utilizing the Morisky Medication Adherence Scale (MMAS-8, Primary outcome) and Antidepressant Side-Effect Checklist (ASEC) to assess both the acceptability and safety of the use of fluoxetine in the patient population [12, 13]. Desire to continue medication/treatment past the 9th month point will also be recorded as will incidence of escalation in psychiatric distress in both patient arms.

#### Aim 2: improvement in symptomology

We will be utilizing the Beck Depression Inventory-II (BDI-II, Primary outcome) [14] Beck Anxiety Inventory (BAI) [15] PTSD Scale-Self report for DSM-5 (PSS-SR5-PTSD) [16]; Pittsburgh Sleep Quality Index (PSQI) [17]; Pain, Enjoyment, General Activity Scale (Peg) [18]; and Patient-Reported Outcomes Measurement Information System-Physical function surveys (PROMIS-PF) [19]. All are validated patient reported surveys. Survey administration will be facilitated by the study team via data entry into portable tablet devices.

### Analysis

#### Sample size determination

**Fig. 3:**
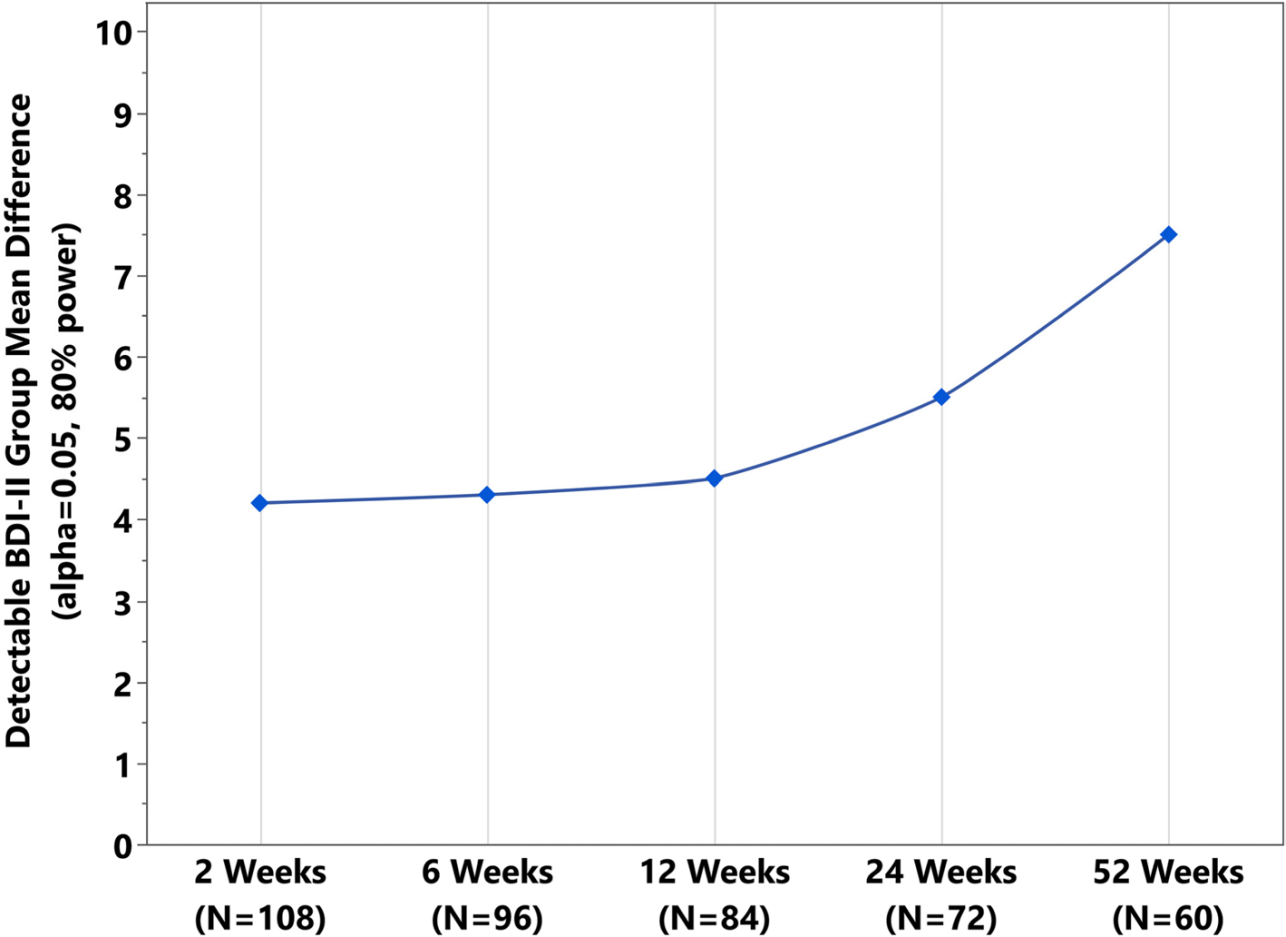
Group mean difference in BDI-II by timepoint

Sample size calculations focused on patient acceptability of intervention (fluoxetine adherence), and secondarily, efficacy of intervention. Calculations assumed 80% power, alpha = 0.05, and a 10% drop rate after baseline; we observed a loss to follow up of approximately 10% at each timepoint with our previous prospective clinical trial [3]. Our goal is to follow the patients for 12 months and with five follow-up time points scheduled; we estimate a dropout rate of 50% by 12 months. Our target sample size is *n* = 90 (*n* = 45 per group); we will aim to recruit *n* = 100 at baseline. For medical adherence (MMAS-8, Aim 1), if we observe 75% of sample with medium or high adherence in *n* = 45 of the fluoxetine group, the 95% confidence interval would be (63%, 88%); with *n* = 23 (potential at 1 year), confidence interval would be (56%, 92%). For longitudinal efficacy analyses (BDI-II, Aim 1), the proposed linear mixed model analysis is able to handle missing data and uneven data structure for patients; all patients will be included in these analyses as long as they have one follow-up timepoint of completed data; GLIMMPSE online program was used for sample size calculation for Aim 1 and estimates were used from our previous study from our group [3]. For Aim 1, *n* = 90 will be able to detect between group differences of 4 points in BDI-II (SD = 9); these analyses included five time points for repeated measures, with assumed correlation of 0.75 for adjacent timepoints that decreases to 0.5 for farthest apart timepoints. Baseline BDI-II was also included in the model, with assumed correlation of 0.55 with 2-week BDI-II that decreases to 0.2 at 1-year BDI-II (Fig. 3).

#### Data analysis plan

Analyses will be performed in JMP Pro 15 (SAS Institute, Inc., Cary, NC, USA). Continuous measures will be summarized as means and standard deviations and categorical measures will be summarized as counts and percentages. For Aim 1, counts and percentages (with 95% confidence intervals) of adverse outcomes will be recorded. Medicine adherence (primary outcome) will be measured at every time point starting at week 6, with patients classified as high adherence (8), medium adherence (6, 7), and low adherence (< 6), which will be reported as percentages with 95% confidence intervals [20]. For intervention efficacy analyses (Aim 2), linear mixed models for repeated measures will be used for both primary (BDI-II) and secondary (BAI, PSQI, PSS-SR5, PEG, PROMIS-PF scales) outcomes. These models account for within-person correlations across time points and can handle uneven data structure. These models utilize maximum likelihood estimation so that patients with incomplete data at some timepoints will still be included in analyses. Baseline measures (if applicable) will be included as independent variables in these models along with intervention group. Tukey’s post hoc test (which adjusts for multiple comparisons), will be used to test for pairwise group differences at each timepoint, with mean differences reported with 95% confidence intervals. A significant group × time interaction will be modeled to test if changes in outcomes differed between the intervention groups. *P* < 0.05 will be considered statistically significant, with only one primary outcome, no correction for multiple comparisons will be performed; raw p-values for all analyses will be reported [21, 22].

#### Data management and confidentiality

Data will be collected using electronic case report forms (CRFs) and immediately entered into the Research Electronic Data Capture (REDCap) database. REDCap is a secure, Web-based software platform designed to support data capture for research studies, providing (1) an intuitive interface for validated data capture; (2) audit trails for tracking data manipulation and export procedures; (3) automated export procedures for seamless data downloads to common statistical packages; and (4) procedures for data integration and interoperability with external sources [23, 24]. All surveys utilized are validated tools.

#### Dissemination policy

During the study, the research team will notify participants of new information that may become available and might affect their decision to remain in the study. They may not be allowed to see the research information collected about them for this research study, including the research information in their medical record, until after the study is completed. When this research study is over, participants will be allowed to see any research information collected and placed in their medical record. A de-identified dataset can also be made available to participants after completion of the study, upon reasonable request to the corresponding author.

## Discussion

For healthcare workers to properly treat the traumatic injuries of a patient, mental health must be included in the process. Increasing research into the impact of mental health on patient recovery has made it evident that healing post-musculoskeletal trauma requires more than physical health interventions. With such severely negative mental health outcomes post-trauma, orthopedic departments must recognize that improving their patient’s quality of life goes beyond physical healing. As previous studies have established, talk therapy and support groups do not provide the level of support necessary to have a strong positive impact on patients with such severe trauma [4]. By administering pharmaceutical interventions to prevent severe depression, anxiety, and other mental health issues, our study goes beyond the norm to help victims of musculoskeletal trauma.

The concern of PTSD in orthopedic trauma is significant enough to already be discussed in previous literature, making the work of our study even more essential. Previous literature has investigated PTSD in orthopedic trauma patients to determine if their described negative mental health symptoms are enough for a PTSD diagnosis [25]. Although the study does recognize the potential connection between physical and mental healing, they do not provide any interventions or treatments for patients’ negative symptomology as ours does [3]. Another study uses paroxetine—another SSRI antidepressant—which is similar to fluoxetine used in our study [26]. However, this study had 50% of participants using paroxetine reporting side effects [26]. After understanding the prior research regarding SSRIs for PTSD in orthopedic trauma and consulting with the two psychiatry members of our team, we decided to conduct our study using fluoxetine instead of paroxetine.

In addition to helping our patients physically and mentally, we are also investigating a potential new standard-of-treatment for trauma survivors. One of the goals of this study is having an orthopedic trauma physician administer psychiatric medications as part of their post-surgical treatment. By normalizing psychiatric medical intervention prescribed by orthopedic surgeons, we can help streamline the process of simultaneous mental and physical health support. If the results of this study determine more positive outcomes for patients randomized to fluoxetine, we can continue to develop treatment strategies incorporating psychiatric medications to the post-orthopedic surgery treatment protocol.

## Future directions

Since the initiation of this trial in Sept 2021, a Cochran Review in February 2022 was released concluding there was “uncertain evidence” for the use of certain pharmacologic interventions in the prevention of PTSD and that “future research might benefit from larger samples, better reporting of side effects” [27]. Multiple patients approached for this study have also raised the concern about treating symptoms that do not yet exist.

The study team has discussed these concerns and feel obligated to pivot in light of this new data. We still feel strongly there is value in early treatment, however. Our prior prospective trial demonstrated that initial BDI-II scores correlated with increased risk for developing prolonged depressive and anxious symptomology [28]. We will utilize this to identify an at-risk population and will revise the trial to target this population.

Trial workflow will be revised to:

- Screening criteria remain the same
- Patients will be consented to take the BDI-II survey in the hospital; those that score higher than 14 (14–19 mild, 20–28 moderate, 29–63 severe) will then be consented for the remaining surveys and randomized. Those scoring less than 14 will be excluded from trial.

Change to randomization:

- The randomization will be stratified by gender but not BDI-II

All patients enrolled in the study to-date will be continued to be treated and followed as described in the original study design above. Those who were enrolled prior to the new inclusion criteria (i.e., those with initial BDI-II less than 14) will be analyzed as a separate group.

## Potential limitations

Potential limitations for this study include gender and age differences, participant loss to follow-up and loss to medication adherence. Previous studies conducted by members of this research team has shown us that men tend to come in more often than women for serious musculoskeletal trauma injuries. This may lead to more ease of recruitment of male than female participants. To prepare for this, we have set goals for recruitment of both sexes, illustrated in our enrollment report above (Table 1). Age differences in orthopedic trauma also present a limitation as patients over the age of 65 are already underrepresented in orthopedic trauma injuries [29]. Since the musculoskeletal injuries that predominate in this population are overwhelmingly fall injuries, we must keep in mind that “high energy trauma” described in the inclusion criteria only accounts for falls that occur from higher than ground level. As we are including participants up to 85 years old, it is possible that there will be few patients above the age of 65 meeting the inclusion criteria for this study. This means that a large majority of patients over the age of 65 may be excluded from the trial since many of their falls occur from ground level. This exclusion should not be an issue for younger patient demographics, as their most common mechanisms of injury are due to motor vehicle accidents and penetrating injuries [29].

Losing participants to follow-up has been documented as a limitation in previous studies run in our department [30]. With time, some participants stop returning to clinic leading to our study team being unable to ask the required follow-up questions. To avoid losing participants to follow-up, we will continue to request multiple phone numbers and emails per patient so we can attempt to contact them through multiple avenues. If we are unable to reach them in person, we will proceed to contact their phone numbers and emails and make sure we ask about any changes in their contact information at each follow up visit. Participant loss to medication adherence is also a risk we may encounter. Our study requires consistent participant-administered medication treatment over the course of several months. Although clinical trial adherence is typically self-reported as over 90%, previous studies of comparing self-report to drug in plasma and urine shows that adherence is much lower [31]. Drug adherence must be properly documented to accurately determine medication effects and decrease variance in the study data. We will be closely monitoring participant drug adherence and withdraw participants as necessary if they are not taking the randomized medication as prescribed through the use of the medication adherence questionnaire. By enrolling up to 100 patients, we will prevent a serious negative impact due to loss to follow-up and drug adherence.

## Lessons learned

As we have begun consenting and following up with patients, our team has already made discoveries about our protocol and our patients. Finding patients to approach has come with certain unexpected challenges. Approaching patients to consent requires an empathetic approach for each individual’s circumstances and we found unique gender differences that differed from our expectations. Following up with patients has taught us how their individual thoughts about mental health and medication use can impact their adherence to protocol and willingness to participate in the study.

### Finding patients to approach

- Exclusion criteria have narrowed our options of people to consent. Though we have a limited number of exclusion criteria, the most common exclusions preventing increased recruitment include ground level falls or patients failing to elevate out of the ICU. These situations are commonly seen in our hospital.

### Approaching patients for the consent process

- When approaching patients, using terms like orthopedic trauma “survivor” instead of “victim” helps focus the discussion on their individual strength instead of the traumatic accident.
- When mentioning PTSD to patients, describing the disorder as a potential side effect of trauma that people such as first responders and military veterans endure helps normalize mental health. This can help frame it as something that “strong” people endure and not a weakness.
- Despite our original thoughts on increased recruitment of males, we have found that men are more likely to decline participation when approached and women are more open to participating in a study with a mental health focus. We found no significant gender differences in recruitment after briefly including a male researcher to approach patients for consent.

### Reasoning of patients who declined to participate

- Prior to beginning this study, the study team acknowledged the difficulties that would be encountered due to the ongoing stigma regarding mental health. Many of the patients that were approached for consent declined due to the chance of being randomized to fluoxetine. Some potential subjects even told our study staff that they “don’t want any of that mental health crap” or they are “not crazy and don’t need to worry about their mental health”. Table 3 below details the enrollment table as of this paper’s submission:

**Table 3:** Patient reasoning for declining to participate

Number of subjects decline	Reason for declining
10	Did not want to be on any additional medication
3	Were opposed to potentially being on fluoxetine (Prozac)
6	Did not want to be involved in any research
5	Did not believe they had/were at risk for mental health issues
2	Had barriers for follow-up
2	Did not provide a reason

### Patient participation

- Patients in more severe accidents often spend more time in the hospital and decline participation due to medication fatigue. Unfortunately, these people are often already increasingly frustrated or exhausted, and may be more likely to benefit from mental health treatment.
- Patients may believe they are mentally well and will not encounter mental health problems in their recovery. If these people do endure mental health struggles, they are more likely to be distressed as these feelings were unexpected.
- We found that patients are more likely to be drawn away from taking fluoxetine even while randomized to it. Mental health medications, particularly Prozac, have a negative stigma associated with them [32]. In some cases, the research team member provides enough information about the medication to dispel a patient’s concerns. However, we believe having a mental health provider, in our case Dr. Barbosa De Faria, answer questions about fluoxetine may be more effective in answering questions and dissipating concerns. This can make the patient more likely to participate in the study and maintain adherence to the medication.
- Some patients have a strong bias for or against one medication or another. Typically, this bias is against fluoxetine. However, there are cases in which patients do recognize their current or potential mental health struggles and would prefer to take fluoxetine. Ironically, we have seen multiple cases when a patient notably favors one medication, and then is randomized to the other. Most notably our female patients hope to be randomized to fluoxetine but are indeed randomized to calcium. When a patient particularly favors or is against a medication, there tends to be a great impact on their likelihood of continuing in the study and adhering to the medication.

## Data Availability

Not applicable.

## Abbreviations

PTSD: Post traumatic stress disorder
SSRI: Selective serotonin reuptake inhibitor
CBT: Cognitive behavioral therapy
SAE: Serious adverse event
CRF: Case Report Form
REDCap: Research Electronic Data Capture
PDMP: Prescription Drug Monitoring Program
UL: Upper intake level
BDI-II: Beck Depression Inventory-II
BAI: Beck Anxiety Inventory
PSS-SR5-PTSD: PTSD Scale-Self report for DSM-5
PSQI: Pittsburgh Sleep Quality Index
PEG: Pain, Enjoyment, General Activity Scale
PROMIS-PF: Patient-Reported Outcomes Measurement Information System-Physical function
MMAS-8: Morisky Medication Adherence Scale
ASEC: Antidepressant Side-Effect Checklist
GLIMMPSE: General linear mixed model power and sample size

## References

[1] Vincent HK, Horodyski M, Vincent KR, Brisbane ST, Sadasivan KK (2015). Psychological distress after orthopedic trauma: prevalence in patients and implications for rehabilitation. PM R.

[2] Mitchell C, https://www.facebook.com/pahowho. PAHO/WHO | Mental health problems are the leading cause of disability worldwide, say experts at PAHO Directing Council side event.: Pan American Health Organization / World Health Organization; 2019. Available from: https://www3.paho.org/hq/index.php?option=com_content&view=article&id=15481:mental-health-problems-are-the-leading-cause-of-disability-worldwide-say-experts-at-paho-directing-council-side-event&Itemid=72565&lang=en. Cited 2021 Aug 10

[3] Zdziarski-Horodyski L, Vasilopoulos T, Horodyski M, Hagen JE, Sadasivan KS, Sharififar S (2020). Can an integrative care approach improve physical function trajectories after orthopaedic trauma? A randomized controlled trial. Clin Orthop.

[4] Burns A, Banerjee S, Morris J, Woodward Y, Baldwin R, Proctor R (2007). Treatment and prevention of depression after surgery for hip fracture in older people: randomized, controlled trials. J Am Geriatr Soc.

[5] Grassi L, Nanni MG, Rodin G, Li M, Caruso R (2018). The use of antidepressants in oncology: a review and practical tips for oncologists. Ann Oncol Off J Eur Soc Med Oncol.

[6] Wakida EK, Talib ZM, Akena D, Okello ES, Kinengyere A, Mindra A (2018). Barriers and facilitators to the integration of mental health services into primary health care: a systematic review. Syst Rev.

[7] Rodriguez JJ, Kohn R (2008). Use of mental health services among disaster survivors. Curr Opin Psychiatry.

[8] Fluoxetine (oral route) description and brand names - Mayo Clinic. Available from: https://www.mayoclinic.org/drugs-supplements/fluoxetine-oral-route/description/drg-20063952. Cited 2021 Aug 10.

[9] Wernicke JF (2004). Safety and side effect profile of fluoxetine. Expert Opin Drug Saf.

[10] Are you getting enough calcium?. Mayo Clinic. Available from: https://www.mayoclinic.org/healthy-lifestyle/nutrition-and-healthy-eating/in-depth/calcium-supplements/art-20047097. Cited 2021 Aug 10.

[11] Penckofer S, Kouba J, Byrn M, Ferrans CE (2010). Vitamin D and depression: where is all the sunshine?. Issues Ment Health Nurs.

[12] Zdziarski-Horodyski L, Horodyski M, Sadasivan KK, Hagen J, Vasilopoulos T, Patrick M (2018). An integrated-delivery-of-care approach to improve patient reported physical function and mental wellbeing after orthopedic trauma: study protocol for a randomized controlled trial. Trials.

[13] Bagayogo IP, Turcios-Wiswe K, Taku K, Peccoralo L, Katz CL (2018). Providing mental health services in the primary care setting: the experiences and perceptions of general practitioners at a New York City Clinic. Psychiatr Q.

[14] Beck AT, Steer RA, Brown GK. Beck depression inventory (BDI-II), vol. 10: San Antonio, Tx: Pearson; 1996.

[15] Beck AT, Epstein N, Brown G, Steer RA (1988). An inventory for measuring clinical anxiety: psychometric properties. J Consult Clin Psychol.

[16] Foa EB, McLean CP, Zang Y, Zhong J, Rauch S, Porter K (2016). Psychometric properties of the posttraumatic stress disorder symptom scale interview for DSM–5 (PSSI–5). Psychol Assess.

[17] Buysse DJ, Reynolds CF, Monk TH, Berman SR, Kupfer DJ (1989). The Pittsburgh sleep quality index: a new instrument for psychiatric practice and research. Psychiatry Res.

[18] Krebs EE, Lorenz KA, Bair MJ, Damush TM, Wu J, Sutherland JM (2009). Development and initial validation of the PEG, a three-item scale assessing pain intensity and interference. J Gen Intern Med.

[19] Cella D, Riley W, Stone A, Rothrock N, Reeve B, Yount S (2010). The Patient-Reported Outcomes Measurement Information System (PROMIS) developed and tested its first wave of adult self-reported health outcome item banks: 2005–2008. J Clin Epidemiol.

[20] Morisky DE, Ang A, Krousel-Wood M, Ward HJ (2008). Predictive validity of a medication adherence measure in an outpatient setting. J Clin Hypertens Greenwich Conn.

[21] Kreidler SM, Muller KE, Grunwald GK, Ringham BM, Coker-Dukowitz ZT, Sakhadeo UR (2013). GLIMMPSE: online power computation for linear models with and without a baseline covariate. J Stat Softw.

[22] Li G, Taljaard M, Van den Heuvel ER, Levine MA, Cook DJ, Wells GA (2017). An introduction to multiplicity issues in clinical trials: the what, why, when and how. Int J Epidemiol.

[23] Harris PA, Taylor R, Thielke R, Payne J, Gonzalez N, Conde JG (2009). Research electronic data capture (REDCap)—a metadata-driven methodology and workflow process for providing translational research informatics support. J Biomed Inform.

[24] Harris PA, Taylor R, Minor BL, Elliott V, Fernandez M, O’Neal L (2019). The REDCap consortium: building an international community of software platform partners. J Biomed Inform.

[25] Starr AJ, Smith WR, Frawley WH, Borer DS, Morgan SJ, Reinert CM (2004). Symptoms of posttraumatic stress disorder after orthopaedic trauma. J Bone Jt Surg-Am.

[26] Borrelli J, Starr A, Downs DL, North CS (2019). Prospective study of the effectiveness of paroxetine on the onset of posttraumatic stress disorder, depression, and health and functional outcomes after trauma. J Orthop Trauma.

[27] Bertolini F, Robertson L, Bisson JI, Meader N, Churchill R, Ostuzzi G, Stein DJ, Williams T, Barbui C (2022). Early pharmacological interventions for universal prevention of post-traumatic stress disorder (PTSD) (review). Cochrane Database Syst Rev.

[28] Sharififar S, Gupta S, Vincent HK, Vasilopoulos T, Zdziarski-Horodyski L, Horodyski M (2020). How soon can we identify at-risk patients: examining initial depressive symptomology and opioid use in musculoskeletal trauma survivors?. Injury.

[29] Peterson BE, Jiwanlal A, Della Rocca GJ, Crist BD (2015). Orthopedic trauma and aging. Geriatr Orthop Surg Rehabil.

[30] Slobogean GP, Sprague S, Wells J, Bhandari M, Rojas A, Program of Randomized Trials to Evaluate Pre-operative Antiseptic Skin Solutions in Orthopaedic Trauma (PREP-IT) Investigators (2020). Effectiveness of iodophor vs chlorhexidine solutions for surgical site infections and unplanned reoperations for patients who underwent fracture repair: the PREP-IT Master Protocol. JAMA Netw Open.

[31] Shiovitz TM, Bain EE, McCann DJ, Skolnick P, Laughren T, Hanina A (2016). Mitigating the effects of nonadherence in clinical trials. J Clin Pharmacol.

[32] Smardon R (2008). ‘I’d rather not take Prozac’: stigma and commodification in antidepressant consumer narratives. Health Interdiscip J Soc Study Health Illn Med.

